# Structured approach with primary and secondary survey for major trauma care: an overview of reviews

**DOI:** 10.1186/s13017-022-00472-6

**Published:** 2023-01-04

**Authors:** Silvia Gianola, Silvia Bargeri, Annalisa Biffi, Stefania Cimbanassi, Daniela D’Angelo, Daniela Coclite, Gabriella Facchinetti, Alice Josephine Fauci, Carla Ferrara, Marco Di Nitto, Antonello Napoletano, Ornella Punzo, Katya Ranzato, Alina Tratsevich, Primiano Iannone, Greta Castellini, Osvaldo Chiara, Italian National Institute of Health guideline working group on Major Trauma

**Affiliations:** 1 Unit of Clinical Epidemiology, IRCCS Istituto Ortopedico Galeazzi, Milan, Italy; 2 National Centre for Healthcare Research and Pharmacoepidemiology,, University of Milano-Bicocca, Milan, Italy; 3 Unit of Biostatistics, Epidemiology and Public Health, Department of Statistics and Quantitative Methods, University of Milano-Bicocca, Milan, Italy; 4 General Surgery and Trauma Team, ASST Grande Ospedale Metropolitano Niguarda, University of Milan, Piazza Ospedale Maggiore 3, 20162 Milan, Italy; 5 Centro Nazionale per l Eccellenza Clinica, laQualità e la Sicurezza delle Cure, Istituto Superiore di Sanità, Rome, Italy; 6 Gruppo MultiMedica, IRCCS MultiMedica, Sesto San Giovanni, Milan, Italy; 7 Dipartimento di Medicina Interna, Azienda USL, Ospedale Maggiore, Largo Nigrisoli 2, 40133 Bologna, Italy

**Keywords:** Systematic reviews, Meta-analysis as topic, Structured approach, Diagnostic imaging, Checklist, Advanced trauma life support care, Emergency medicine, GRADE approach

## Abstract

**Background:**

A structured approach involves systematic management of trauma patients. We aim to conduct an overview of reviews about the clinical efficacy and safety of structured approach (i.e., primary and secondary survey) by guideline checklist compared to non-structured approach (i.e. clinical examination); moreover, routine screening whole-body computer tomography (WBCT) was compared to non-routine WBCT in patients with suspected major trauma.

**Methods:**

We systematically searched MEDLINE (PubMed), EMBASE and Cochrane Database of Systematic Reviews up to 3 May 2022. Systematic reviews (SRs) that investigated the use of a structured approach compared to a non-structured approach were eligible. Two authors independently extracted data, managed the overlapping of primary studies belonging to the included SRs and calculated the corrected covered area (CCA). The certainty of evidence was assessed using the Grading of Recommendations Assessment, Development and Evaluation (GRADE) methodology.

**Results:**

We included nine SRs investigating two comparisons in stable trauma patients: structured approach vs non-structured approach (*n* = 1) and routine WBCT vs non-routine WBCT (*n* = 8). The overlap of included primary studies was generally high across outcomes (CCA ranged between 20.85 and 42.86%) with some discrepancies in the directions of effects across reviews. The application of a structured approach by checklist may improve adherence to guidelines (e.g. Advanced Trauma Life Support) during resuscitation and might lead to a reduction in mortality among severely injured patients as compared to clinical examination (Adjusted OR 0.51; 95% CI 0.30–0.89; *p* = 0.018; low certainty of evidence). The use of routine WBCT seems to offer little to no effects in reducing mortality and time spent in emergency room or department, whereas non-routine WBCT seems to offer little to no effects in reducing radiation dose, intensive care unit length of stay (LOS) and hospital LOS (low-to-moderate certainty of evidence).

**Conclusions:**

The application of structured approach by checklist during trauma resuscitation may improve patient- and process-related outcomes. Including non-routine WBCT seems to offer the best trade-offs between benefits and harm. Clinicians should consider these findings in the light of their clinical context, the volume of patients in their facilities, the need for time management, and costs.

**Supplementary Information:**

The online version contains supplementary material available at 10.1186/s13017-022-00472-6.

## Introduction

Trauma injuries account for 4.5 million deaths globally [1] and approximately 530 000 deaths in Europe every year [2]. Almost 10% of the global burden of disease is due to trauma, and trauma is the top contributor to the burden of disease in children and adults aged 10–49 years [3].

Accurate diagnosis is essential for the best therapeutic process. Trauma care is time-sensitive, and early management of life-threatening or limb-threatening conditions is critical. The complexity of the diagnostic process of trauma patients depends not only on its time-dependent nature but it is also due to the numerous settings of care in which diagnosis occurs, the potential existence of multiple injuries in different body areas and the involvement of different specialists within a single diagnostic process [4]. Alongside the diagnostic process, it is also essential to establish adequate management of trauma patients, which represents another critical step that adds complexity to trauma cases. In general, the management of trauma patients can be performed through a structured or a non-structured approach.

The structured approach involves the management of the trauma patient in a systematic manner. For trauma patients, several checklists based on a structured approach have been developed to improve the early management, such as the life support checklist explored in training programmes for trauma assessment and treatment [5–7]. The first training programmes were introduced by the American College of Surgeons with a worldwide spread in the past 20 years: Prehospital Trauma Care (PTC) and Advanced Trauma Life Support (ATLS^®^) [8]. Moreover, a branch of the ATLS programme was established also for nurses, the Advanced Trauma Course for Nurses (ATCN^®^) [9]. In Europe, in 2008 a new European Trauma Course (ETC^®^) was organized, with the non-technical skills of the trauma team as the main objective [10]. Within all of these programmes, there is a comprehensive evaluation of the trauma patient, followed by accurate diagnostic imaging.

Conventionally after hospital admission, the trauma team performs a clinical examination, laboratory examinations and emergency room diagnostic imaging, extended focussed assessment sonography for trauma (eFAST) and chest and pelvis X-ray (primary survey). After this initial evaluation and stabilization, the team performs a secondary survey whose goal is to make a definitive and complete diagnosis of all the injuries produced by trauma (secondary survey). Contrast-enhanced Computed Tomography (CT) can be selectively obtained in patients with evidence or suspicion of some abnormality. Alternatively, thanks to the availability of even faster devices in the proximity of the emergency room, a whole body contrast-enhanced CT scan (WBCT) has been suggested as a non-selective imaging to screen all patients with suspected major trauma, even with borderline vital signs [11, 12].

Although the structured approach is widely used worldwide, the real advantages over a non-structured approach in terms of clinical safety and cost-effectiveness are still not well established. Therefore, we aimed to systematically review the literature to determine the effects of the application of a structured approach and of different diagnostic imaging strategies on critical outcomes during patient trauma resuscitation, in the context of the development of the Italian National Institute of Health guidelines on major trauma-integrated management. We aim to investigate the clinical efficacy and safety of structured approach (primary and secondary survey) by guideline checklist compared to non-structured approach (i.e. clinical examination). Moreover, routine WBCT screening was compared to non-routine WBCT in patients with suspected major trauma.

## Methods

### Study design and setting

This is an overview of reviews conducted following the Cochrane Guidelines [13] to support the major trauma-integrated management guideline panel of the Italian National Institute of Health (Istituto Superiore di Sanità) in formulating recommendations. We followed the Grading of Recommendations Assessment, Development and Evaluation (GRADE)-ADOLOPMENT methodology [14] and the standards defined by the Sistema Nazionale Linee Guida (SNLG) [15].

We followed the reporting guideline for an overview of reviews of healthcare interventions PRIOR [16, 17]. The protocol of the present overview of reviews is stored at the following link: https://osf.io/2s68r/.

### Eligibility criteria

According to Cochrane’s definition, a systematic review (SR) is a review of the literature in which one “attempts to identify, appraise and synthesize all the empirical evidence that meets pre-specified eligibility criteria to answer a specific research question by using explicit, systematic methods that are selected with a view aimed at minimizing bias, to produce more reliable findings to inform decision-making” [18]. We included SRs if they met the following criteria: (1) *population*: children, young people and adults experiencing major trauma; (2) *interventions*: structured approach (primary and secondary survey) following guidelines such as Advanced Trauma Life Support (ATLS) or European Trauma Course (ETC) or sequence Airway, Breathing, Circulation, Disability, Exposure (ABCDE) compared to non-structured approach (e.g. clinical examination without a predefined sequence and diagnostic examinations as needed); moreover routine WBCT screening was compared to non-routine WBCT. Patients in non-routine WBCT may receive after initial evaluation and standards radiological examinations, CT scans of the selected district (e.g. C-spine) or WBCT as needed; (3) *setting*: in-hospital, emergency department (ED) and emergency room (ER) resuscitation phase. Studies including patients with trauma resulting from burns were excluded.

### Outcome measures

Primary outcomes selected for the analyses were: overall mortality, mortality at 24 h, intensive care unit (ICU) admission/ICU length of stay (LOS), complications (e.g. multiple organ failure [MOF], multiple organ dysfunction syndrome [MODS], missed injuries), adherence and disability. Secondary outcomes were: hospital admission/ hospital LOS, time spent in Emergency Department (ED), time spent in Emergency Room (ER) and radiation dose. Outcomes were prioritized by the panel of the Italian National Institute of Health within the major trauma-integrated management guideline, Additional file 1: Appendix A. In addition, as an important domain of the GRADE approach, costs were included.

### Search strategy

We searched the following electronic databases: MEDLINE (PubMed), EMBASE (Elsevier, EMBASE.com) and Cochrane Database of Systematic Reviews up to 3 May 2022 with language restricted to English, Italian, Spanish, French and German. We checked the reference lists of all studies included. The search strategy is outlined in Additional file 1: Appendix B.

### Study selection and data extraction

Two independent authors screened titles and abstracts according to the eligibility criteria. Following the first phase, they independently assessed the full text of potentially relevant studies for inclusion. Any disagreement was solved by a discussion with one of the authors. A standardized data collection form was used to extract the following information: (i) SR characteristics, (ii) Patient, Intervention, Comparison and Outcome (PICO) questions, and (iii) outcome data. The authors of the selected studies were contacted if the study data were not reported in detail or were incomplete.

### Internal validity and certainty of the evidence

We used the Assessing of Methodological quality of Systematic Reviews 2 (AMSTAR 2) for assessing SRs [19].

The certainty of evidence (CoE) of each outcome was judged through five dimensions (risk of bias, consistency of effect, imprecision, indirectness and publication bias) by the GRADE approach [20]. The evidence was downgraded from “high quality” by one level if serious, or by two levels if very serious limitations were found for each of the five above-mentioned dimensions. We presented a summary of findings describing the treatment effects, the CoE and the reasons for limitations.

In order to consider more reviews, a GRADE algorithm, developed for Cochrane overviews of reviews, was used to ascertain the strength of evidence of the reviews included in each treatment comparison for all primary outcomes. In this algorithm, each review starts with a ranking of high certainty and is downgraded:

- by 1 level for serious methodological concerns such as sample size between 100 and 199 participants; high risk of bias in randomization and blinding for > 75% of included studies; high heterogeneity (*I*^2^ > 75%); and “No” on one of these AMSTAR 2 items: a priori research design, comprehensive literature search, duplicate study selection, or duplicate data extraction
- by 2 levels for very serious concerns such as sample size < 100 participants and “No” on two or more of these AMSTAR 2 items: a priori research design, comprehensive literature search, duplicate study selection, or duplicate data extraction [21].

## Data synthesis

We followed the methodology outlined in the Cochrane Handbook’s chapter on overviews of reviews [22] and the decision tree proposed by Hennessy et al. [23] to interpret the results providing context for clinical implications.

### Managing overlapping

We provided a list of the primary studies included in each SR that were collated and cross-referenced in a matrix of evidence table to ascertain the degree of overlap between reviews. We calculated the corrected cover area (CCA) for the overall sample of SRs according to different comparisons stratified by outcomes. We examined the matrix for each outcome providing an interpretation of overlapping: “slight” (CCA 0–5%), “moderate” (CCA 6–10%), “high” (CCA 11–15%) or “very high” (CCA > 15%) [24].

### Summary of evidence

We provided a narrative synthesis of the characteristics of the included SRs. We presented outcome data in results tables reporting effect size (e.g. mean difference [MD], standardized mean difference [SMD], relative risk [RR], odds ratio [ORs]) and their 95% CI, the number of studies and participants, treatment comparison and CoE. For the primary outcome, a visual map of the scientific evidence was created to visually display the information of each review [25]. The graphic display of the mapping is based on bubble plots, where each bubble represents one SR. This graphic provides information in three dimensions: (1) on the *y*-axis there is the rating of authors’ conclusions as “beneficial for intervention”, “no effect” and “beneficial for control” (they were further described in the data extraction section); (2) on the *x*-axis, the GRADE assessment is shown; and (3) we displayed the bubble size proportionally to the number of participants included in each SR.

We interpreted results using a conceptual framework presenting discordant results by comparisons for each outcome [26, 27]. Specifically, we examined the concordance or discordance of results in terms of different directions of the effects (e.g. effective interventions, ineffective interventions, no differences [13]) and explored the sources of heterogeneity. To assess discordances in the direction, we moved from a model in which measures of association are lower or higher than 1.0 for dichotomous outcomes, or 0 for continuous outcomes (i.e. OR of 0.80 is a favourable profile for the intervention group reducing overall mortality).

Statistical significance is set at *p* < 0.05. All tests will be two-sided. Data analysis was performed with STATA software.

### Decision tree for overlapping reviews analysis

A decision tree was applied in case of discordant results and/or very high overlapping of included systematic reviews addressing the same PICO question, in order to avoid time-consuming and repetition of the same information (i.e. double counting of primary studies). Only the best systematic review with (i) the highest quality of evidence, (ii) the most updated bibliographic search, and (iii) a large covered area (i.e. overlapping) of included primary studies was considered for each outcome of interest in order to hypothesize the implication for practice.

## Results

### Study selection

**Fig. 1:**
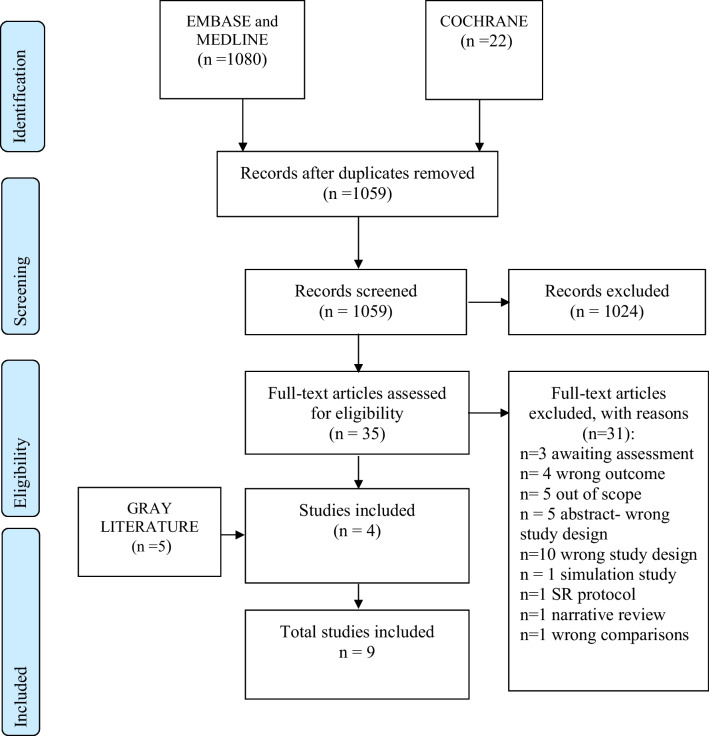
Study selection

After the removal of duplicates, 1059 records were retrieved and 35 records were assessed for eligibility. Finally, nine SRs were included. The flow diagram is shown in Fig. 1.

### General characteristics

**Fig. 2:**
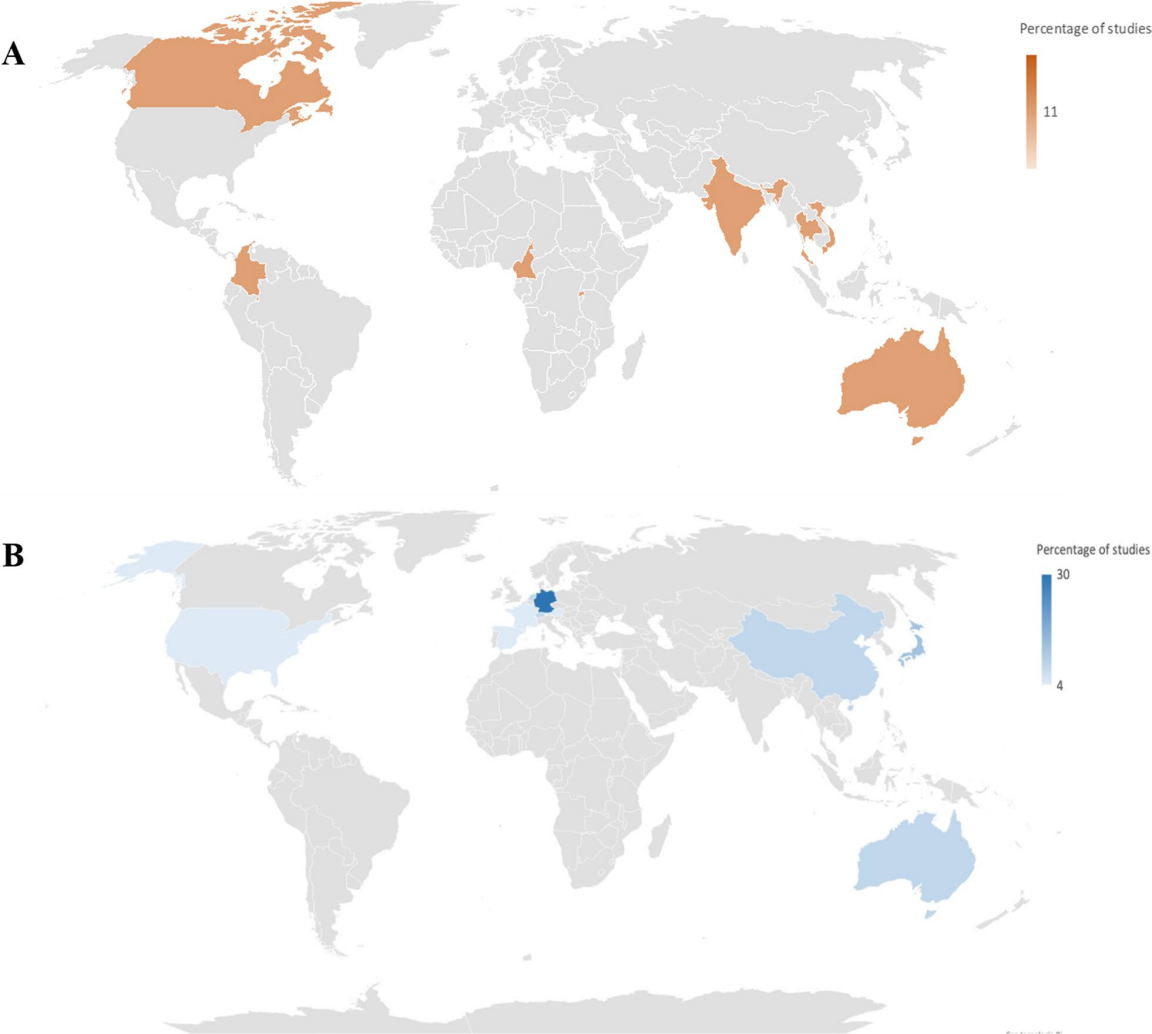
Countries of primary studies included in **A** structured approach vs non-structured approach; **B** routine WBCT vs non-routine WBCT

The included reviews were conducted between 2012 and 2020 with one-third (33.33%) published in the past 5 years (i.e. after 2017). Literature search dates for the included reviews ranged from 2010 to 2019. Across SRs a total of 22 unique primary studies were found. Additional file 1: Appendix C reported study characteristics. A median of 7 primary studies (Interquartile range [IQR] 3–11) were included. Non-randomized controlled trials were the most common design of primary studies (*n* = 19) followed by controlled before-and-after studies (*n* = 2), and randomized controlled trials (RCTs) (*n* = 1). We categorized the two following comparisons: (1) “structured approach vs non-structured approach” (*n* = 1) and (2) “routine WBCT (always in all patients with suspected major trauma) vs non-routine WBCT (selective scans in selected patients on the basis of emergency room tests)” (*n* = 8). The first comparison included studies investigating trauma in adults and children carried out mainly in America, Asia and Australia. The second comparison included studies investigating trauma only in adults carried out mainly in North America, Europe, Asia and Australia (Fig. 2).

The general characteristics are reported in Table 1. The overall mortality was the most investigated outcome (9 SRs, 100%). We mapped the frequency of the outcomes across all included SRs in Table 2.

**Table 1:** General characteristics

SR author year	Data search	Type of included studies	Population	Description interventions/comparison
van Maarseveen (2020)	January 2019	2 studies: Controlled before-and-after study	Adults and paediatrics trauma patients	“Application of a checklist versus no application” comparison 1: structured approach/ clinical examination
Arruzza (2020)	December 2019	15 studies: 14 cohort studies and one RCT	“Trauma patients” adults	“WBCT as part of the primary survey vs conventional radiological procedures” comparison 2: routine WBCT/ non-routine WBCT
Chidambaram (2017)	September 2016	11 studies: 5 prospective cohort studies, 5 retrospective studies and 1 RCT	“Trauma patients”; studies with paediatric patients were excluded	“conventional selective imaging vs whole body CT (WBCT) scanning” comparison 2: routine WBCT/ non-routine WBCT
Hajibandeh (2015)	October 2013	9 studies: 8 retrospective and 1 prospective cohort studies	“Trauma patients” adults	“WBCT vs non-WBCT (i.e. conventional imaging)” comparison 2: routine WBCT/ non-routine WBCT
Van Vugt (2013)	May 2013	0 studies	People who had sustained all types of blunt high-energy trauma (including blast or barotrauma)	resuscitation algorithms using routine CT vs algorithms using selective CT comparison 2: routine WBCT/ non-routine WBCT
Caputo (2013)	2013	7 studies: cohort studies	“Human studies”, adults	“WBCT vs selective scan” comparison 2: routine WBCT/ non-routine WBCT
Jiang (2013)	December 2013	11 studies: cohort studies	SR: adult blunt MTPs (age > 16 years, injury severity score (ISS) > 16)	WBCT vs selective radiological imaging protocol (X-ray of the pelvis and chest, trans-abdominal sonography, and/or selective CT) comparison 2: routine WBCT/ non-routine WBCT
Healy (2013)	October 2012	6 studies: observational studies	SR: “Trauma patients”; studies were excluded if patients were predominantly under 16 years of age	“WBCT vs selective CT scanning” comparison 2: routine WBCT/ non-routine WBCT
Sierink (2012)	November 2010	4 studies: cohort studies	SR: Studies with a mainly adult study population were included (defined as median age of the study group above 16 years)	“immediate WBCT vs conventional imaging and selective CT” comparison 2: routine WBCT/ non-routine WBCT

**Table 2:**
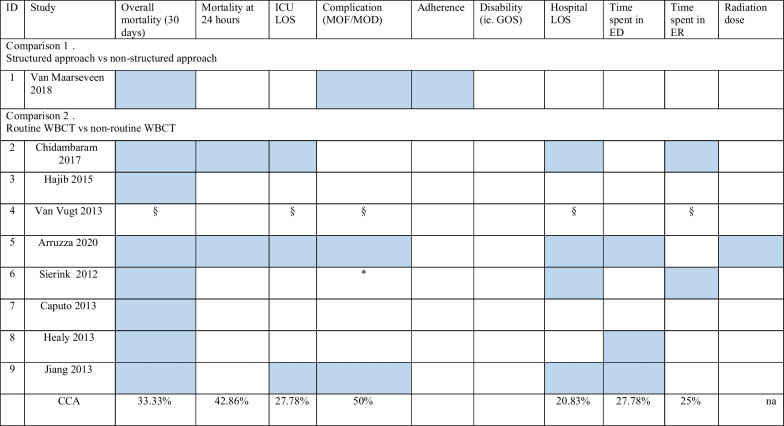
Mapping outcomes across included SRs

*CCA* corrected covered area; *GOS* Glasgow Outcome Scale

§The SR planned to investigate overall mortality (30-day survival), adverse events, time spent in the ER, Hospital LOS, ICU LOS in RCTs but no studies have been found

*The SR planned to investigate the complication but none of the included studies reported the outcome

### Internal validity and quality of evidence

All SRs were judged to be critically low with AMSTAR 2 (Additional file 1: Appendix D). The certainty of evidence in comparison 1 (structured approach vs non-structured approach) was very low across all investigated outcomes (Additional file 1: Appendix E). The certainty of evidence in comparison 2 (routine WBCT vs non-routine WBCT) ranged from very low to moderate with the GRADE approach (Additional file 1: Appendix E). We mainly downgraded the evidence for risk of bias in included studies and imprecision of the estimates. In most analyses, confidence intervals crossed the line of no difference with plausible effects in favour of the experimental group.

### Overlapping and summary of evidence

#### Comparison 1. Structured approach vs non-structured approach

##### Overall mortality

One SR [28] (including one study in adult population) investigated this outcome reporting no difference in odds of mortality in the overall study sample (OR 1.02; CI 0.77–1.34 *p* = 0.904), whereas there was a statistically significant mortality reduction of 50% among patients with the most severe injuries (Injury Severity Score > 25) (adjusted OR [aOR] 0.51; 95% CI 0.30–0.89; *p* = 0.018).

##### Adherence to structured approach

One SR [28] including two studies reported this outcome without performing a meta-analysis. In one study in the paediatric setting, 14 of the 30 ATLS tasks were completed by doctors more often after checklist introduction (for all *p* ≤ 0.01). After adjustment, the ORs were 2.66 (95% CI 2.07–3.42) and 2.46 (95% CI 2.04–2.98) times higher for completing, respectively, primary survey, and secondary survey tasks, after the introduction of a checklist. In the other study in the adult population, 18 of the 19 clinical tasks were significantly (*p* < 0.05) more frequently performed after the implementation of the World Health Organization (WHO) trauma care checklist.

##### Complications

One SR [28] including one study reported this outcome in the adult population. The incidence of one of the ten complications (pneumonia) was slightly higher after the introduction of the checklist (aOR 1.69, 95% CI 1.03–2.80). The aOR for the other nine complications was not significantly different.

##### Complications as missed injuries

One SR [28] including one study reported this outcome in 3422 adult trauma patients: incidence of missed injuries did not differ before and implementation of a checklist (aOR 0.62; 95% CI 0.19–2.03; p = 0.437).

#### Comparison 2. Routine WBCT vs non-routine WBCT

##### Overall mortality

**Fig. 3:**
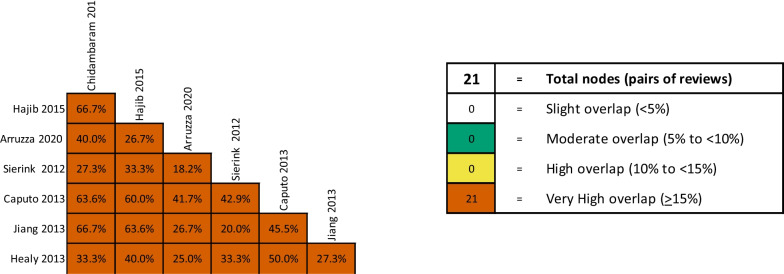
Overlapping–overall mortality

Seven SRs reported the overall mortality outcome [29–35], and one SR did not report any results because it did not include any eligible study [36]. The CCA showed a very high overlapping of citation primary studies (33%, Fig. 3). Compared to non-routine WBCT, routine WBCT was effective in reducing mortality in all SRs (concordant results), except for three SRs [29, 33, 35] that found no difference between groups.

##### 24-h mortality

Two SRs with a very high overlapping of citation primary studies (42.86%) reported mortality at 24 h with discordant results: one SR showed statistically significant results favouring routine WBCT and the other no difference between groups [29, 34].

##### ICU LOS

Three SRs investigated ICU LOS [29, 31, 34] with a very high overlapping of citation primary studies (27.78%). There are no differences between routine WBCT and non-routine WBCT in two SRs [29, 31], whereas non-routine WBCT was effective compared to routine WBCT for reducing ICU LOS in one SR [34].

##### Complications

Two SRs investigated complications, reported as MODS or MOF [29, 31], with a very high overlapping of citation primary studies (50%). With concordant results, there are no differences between routine WBCT and non-routine WBCT in both SRs [29, 31].

##### Radiation dose

One SR [29] reported a statistically significant reduction in dose with non-routine WBCT.

##### Hospital LOS

Four SRs reported the hospital LOS [29, 31, 33, 34] with very high overlapping of citation primary studies (20.83%). No differences between routine WBCT and non-routine WBCT were reported in all SRs except for one SR [34] reporting non-routine WBCT as more effective than routine WBCT to reduce hospital LOS.

##### Time spent in ER

Two SRs reported this outcome of interest [33, 34] with a very high overlapping of citation primary studies (25%). In one SR, routine WBCT was effective compared to non-routine WBCT [34] to reduce time spent in ER. The other SR [33] did not perform a meta-analysis due to the heterogeneity of measurements.

##### Time spent in ED

Three SRs reported this outcome [29, 31, 35] with a very high overlapping of citation primary studies (27.78%). All SRs [29, 31, 35] showed concordant results: routine WBCT compared to non-routine WBCT was effective in reducing time spent in ED.

### Hospital costs

One SR [29] including three studies investigated hospital stay cost-based parameters. Two primary studies [37, 38] conducted in Taiwan and Europe discovered that the costs associated were not statistically significant between routine WBCT and non-routine WBCT. Particularly, data reported from a multicentre RCT showed that the hospital costs of European hospital stay were €24 967 (95% CI 21 880–28 752) for the WBCT group and €26 995 (23 326–30 908) for the standard work-up group (*p* = 0.44). Alternatively, another primary study [39] conducted in North America found that the mean cost of a blunt trauma patient’s hospital stay increased by $4 971 after the WBCT protocol was introduced.

### Decision tree for overlapping reviews analysis

In the comparison “routine WBCT versus non-routine WBCT”, SRs showed discordant results in the presence of very high overlapping of primary studies (33.33%) for the primary outcome of overall mortality. Thus, a decision tree was applied to guide the interpretation of findings.

**Fig. 4:**
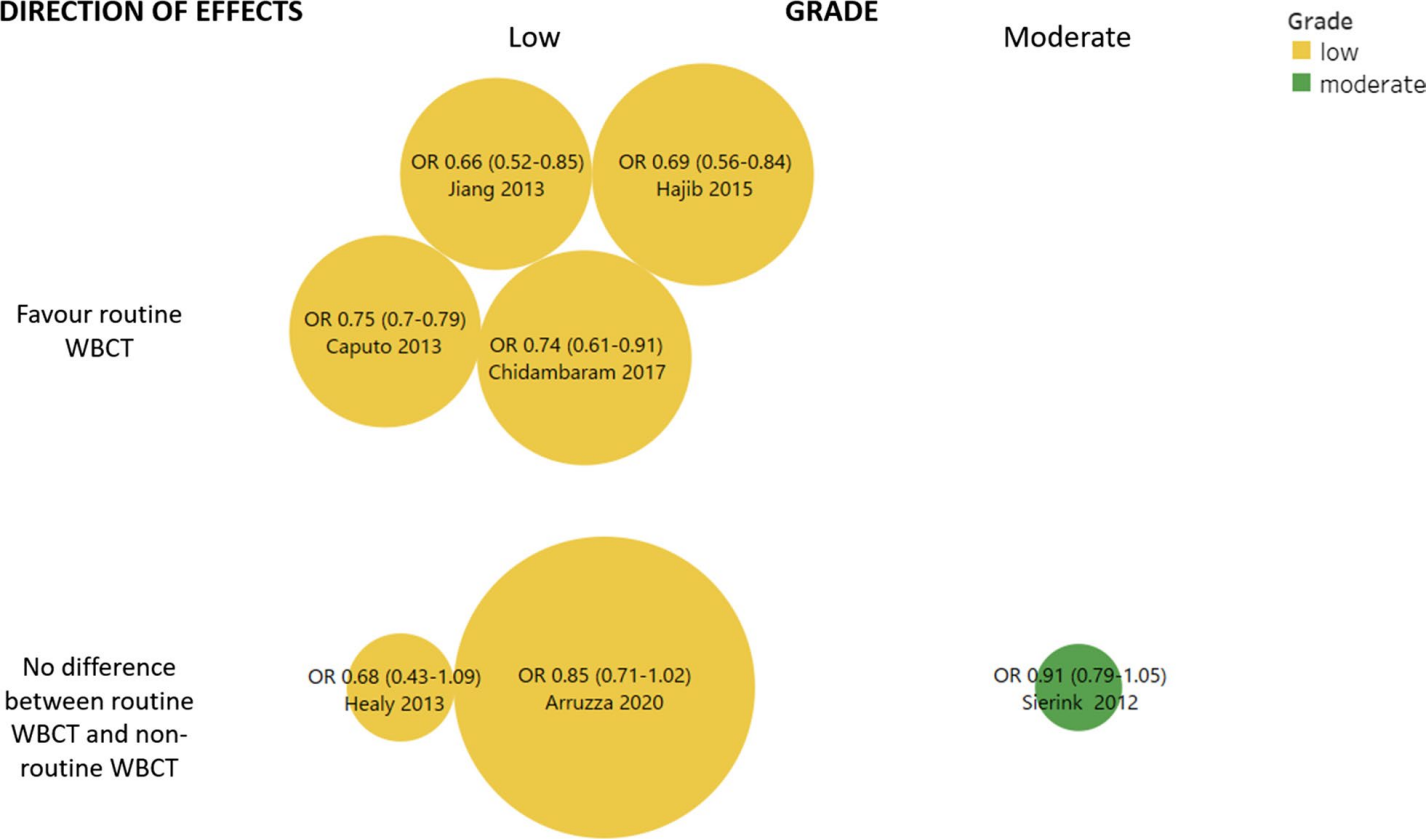
Evidence map for overall mortality

The most updated SR was Arruzza et al. [29] including 63 539 patients: with low certainty of the evidence, mortality showed a non-statistically significant difference between groups (OR 0.85, 95% CI 0.71–1.02). Accordingly, a similar direction of effect was first reported by Sierink [33] with moderate certainty of evidence (OR 0.91, 95% CI 0.79–1.05). Figure 4 shows the evidence map of overall mortality linking the direction of the effects and the certainty of the evidence. Considering all outcomes, routine WBCT seems to offer little to no effects in reducing mortality and time spent in ER or ED, whereas non-routine WBCT seems to offer little to no effects in reducing radiation dose, ICU LOS and hospital LOS (low-to-moderate certainty of evidence). In Table 3, we reported the evidence for all outcomes for routine WBCT versus non-routine WBCT.

**Table 3:** Evidence of routine WBCT versus non-routine WCBT

COMPARISON routine WBCT vs non-routine WBCT	Overlapping CCA	Certainty of evidence (GRADE)	Update search strategy	Overall effects	Total participants	Concordance/Discordance
*Overall mortality*						
Chidambaram (2017)	33.33%	LOW	September 2016	OR 0.74 (0.61,0.91)	32,207	Favour routine WBCT
Hajibandeh (2015)		LOW	October 2013	OR 0.69 (0.56,0.84)	34,468	Favour routine WBCT
Arruzza (2020)		LOW	December 2019	OR 0.85 (0.71,1.02)	63,539	No difference
Sierink (2012)		MODERATE	November 2010	OR 0.91 (0.79,1.05)	5309	No difference
Caputo (2013)		LOW	2013	OR 0.75 (0.7,0.79)	25,782	Favour routine WBCT
Jiang (2013)		LOW	December 2013	OR 0.66 (0.52,0.85)	26,065	Favour routine WBCT
Healy (2013)		LOW	October 2012	OR 0.68 (0.43,1.09)	8180	No difference
*24-h mortality*						
Chidambaram (2017)	42.86%	LOW	September 2016	OR 0.72 (0.66,0.79)	20,206	Favour routine WBCT
Arruzza (2020)		MODERATE		OR 0.86 (0.65,1.21)	20,374	No difference
*ICU LOS*						
Chidambaram (2017)	27.78%	LOW	September 2016	MD 1.97 (1.59, 2.34)	22,631	Favour non-routine WBCT
Arruzza (2020)		LOW	December 2019	SMD 0.08 (− 0.13,0.29)	18,645	No difference
Jiang (2013)		LOW	December 2013	MD 0.95 (− 0.08,1.98)	18,755	No difference
*Complication (MODS/MOF)*						
Arruzza (2020)	50%	LOW	December 2019	OR 1.88 (0.61,5.83)	18,010	No difference
Jiang (2013)		LOW	December 2013	OR 2.50 (0.82,7.65)	17,763	No difference
*Radiation dose*						
Arruzza (2020)	NA	MODERATE	December 2019	No meta-analysis: all 4 included studies reported *p* < 0.001	3029	Favour non-routine WBCT
*Hospital LOS*						
Chidambaram (2017)	20.83%	MODERATE	September 2016	MD1.03 (0.25,1.81)	21,710	Favour non-routine WBCT
Sierink (2012)		MODERATE	November 2010	MD 0.57 (− 5.84, 6.98)	4991	No difference
Arruzza (2020)		LOW	December 2019	SMD 0.08 (− 0.18, 0.34)	18,528	No difference
Jiang (2013)		MODERATE	December 2013	MD 0.56 (− 0.03, 1.15)	17,393	No difference
*Time spent in ER*						
Chidambaram (2017)	25%	LOW	September 2016	MD − 14.18 (− 17.02,− 12.60)	5912	Favour routine WBCT
Sierink (2012)		NA	November 2010	No meta-analysis		–
*Time spent in ED*						
Arruzza (2020)	27.78%	LOW	December 2019	SMD − 0.70 (− 1.19, − 0.22)	18,789	Favour routine WBCT
Healy (2013)		LOW	October 2012	SMD − 32.39 (− 51.78, − 13.00)	6073	Favour routine WBCT
Jiang (2013)		LOW	December 2013	SMD − 25.58 (− 43.04, − 12.12)	18,294	Favour routine WBCT

## Discussion

This comprehensive overview of reviews included nine systematic reviews representing 22 unique primary studies and offers positive clinical results with very low to moderate-high evidence in patients with trauma.

The application of a structured approach by checklist may improve adherence to guidelines and workflow (e.g. ATLS) during resuscitation and might lead to a reduction in mortality among severely injured patients as compared to non-structured approach. The structured approach starts with the primary evaluation, which includes a classic sequence of clinical (e.g. ABCDE) and instrumental (i.e., eFAST, chest and pelvis X-ray) investigations aimed at verifying the existence of life-threatening conditions which need immediate correction. It is well recognized that this is the key to reducing mortality in severely injured patients with unstable vital signs [40]. This approach has been popularized in North America by the American College of Surgeons at the end of the twentieth century and quickly spread all over the world [8, 9, 41]. Interestingly, in our SRs no primary study was found from European countries of either the ATLS or the ETC courses about the improvement in trauma care. Probably, ATLS approach to trauma was diffused in Europe when there was already a general acceptance of the ATLS method, while ETC did not have such a diffusion to allow for significant evaluations.

After primary evaluation and stabilization of vital signs, if needed, the secondary evaluation (clinical history, head-to-toe physical examination) aims to obtain a definitive diagnosis of all the injuries produced by trauma. The routine WBCT has the undoubted advantage of high diagnostic accuracy for almost all types of post-trauma injuries [42]. Surprisingly, in our SRs there is little to no evidence of the effectiveness of routine WBCT across multiple outcomes reported in more than one review. Even if routine WBCT reduces time in ED and ER, it does not seem to reduce the mortality rate and complications, while it involves more radiation doses than non-routine WBCT. Moreover, a preliminary hospital cost analysis showed that there were no differences between WBCT and non-routine WBCT in two out of three primary studies. However, this can depend on different management of sources across countries.

The major dispute is on overall mortality and 24-h mortality rates showing discordant results across SRs. During the past years, several studies have shown an association between routine WBCT scanning and survival in patients with trauma: in some hospitals, especially in Germany, the emergency room has been organized with a CT scan device inside and the trauma patient receives a total-body scan during initial resuscitation. This approach has been associated with an improved outcome, but it has been investigated only in retrospective observational studies [11]. Furthermore, the availability of a CT scan in the emergency room is rare and could be associated with the overuse of this imaging. Discrepancies between SRs may be due to different included primary studies. The most recent SR [29] found no differences between routine WBCT and non-routine WBCT. This SR included the unique RCT [38] (multicentre, including 1 403 patients) published in 2016 which demonstrated no difference between groups, but did not include the retrospective study of Wada et al. [43], which demonstrated significant favourability towards routine WBCT. However, Wada et al. study can be at risk of some selection bias: i) CT performed at the discretion of the attending physician based on individual patient condition and not according to a predefined protocol, ii) the small sample size in non-routine WBCT group (20 vs 132) compared to routine WBCT and iii) significant differences in baseline characteristics (e.g. injury severity score, systolic blood pressure, revised trauma score) between groups. In fact, patients included in the non-routine WBCT group were probably less severely injured and consequently underwent a shorter hospital stay [43].

With concordance of all SRs, no differences between routine WBCT and non-routine WBCT on complication (e.g. MOF and MODS) were found. There is little evidence of the effectiveness of routine WBCT in reducing time spent in ED and ER but probably an increase in radiation dose. These findings have implications entailing faster diagnosis time for definitive treatment and lessening the impact of ED overcrowding but it is certainly also possible that routine WBCT scanning may do more harm than good (e.g. cancer rates, contrast-induced nephropathy). The need to limit the radiation dose is another important factor in deciding which patients might benefit from an immediate WBCT scan [38] with important implications not only from an ethical perspective but also from broader societal and national health care policy point of view.

Another condition associated with the futile use of routine WBCT could be the high rate of over-triage in trauma patients admitted after a high-energy impact and with normal vital signs, which can be as high as 75%. Modern protective devices (pre-tensioned seatbelts, airbags, helmets and car bodies with progressive deformation) have reduced the severity of injuries and many patients with high-energy impact show only minor injuries. Indiscriminate use of WBCT in these patients could lead to a significant “over-scanning”. Emergency room tests and 6–8 h of observation, followed by a careful re-evaluation, can select patients who need WBCT and discharge those with no injuries [44].

### Implication for future

We found that the use of checklist for a structured approach is not investigated in Europe; in fact, primary studies were mainly carried out in America, Asia and Australia. This can reflect the clinical practice, where differences between countries can impact the use of structured approaches, as well as for the use of routine WBCT versus non-routine WBCT. In some countries, the use of structured approach is already a standard of care (e.g. North America) but not in others (e.g. Europe) [45–48].

Future studies across countries should assess the introduction of a structured approach by using a checklist to promote its adoption in trauma care as a tool for changing clinical practice, to increase the robustness of scientific evidence and to guarantee equity in healthcare assistance. Our findings support the use of structured approaches including the use of non-routine WBCT after initial assessment in selected patients.

Because of the increased radiation dose and possible other harms (e.g. induced malignancies), future research should focus on the selection of patients who would benefit from immediate total-body CT.

As well, more RCTs should be conducted to confirm preliminary results. RCTs should explore impacts on more specific patient subgroups and other relevant outcomes such as cost-effectiveness, complication rates, diagnostic accuracy and missed injury rates. Such exploration will inform a more holistic perspective regarding the efficacy of WBCT within the clinical environment.

### Strength and limitations

This is the first overview of reviews evaluating the clinical implications using a structured versus non-structured approach. However, some limitations must be taken into account. Firstly, we considered SRs including one RCT and 21observational studies. The retrospective nature of some studies introduces biases otherwise minimized in prospective cohorts or RCTs. Only one RCT was considered in two SRs and it was inappropriately combined with other observational studies in the same meta-analysis [29, 34]. Secondarily, the control group encompassed all non-routine WBCT-related protocols (both ATLS and strictly selective CT), as the definition of “conventional” varies among countries and even between institutions within the same region. Third, in the first comparison, one SR included both adult and children population reporting separate results according to a specific population [28], whereas in the second comparison the paediatric field was not assessed and we were aware that the management of injuries (including the use of imaging and different modalities) is not the same. Thus, these findings cannot be generalized to children. Fourth, we planned to investigate other primary and secondary outcomes than mortality, but scarce literature is at the moment available. For instance, missed injury rates, as part of complications, were reported only in one study in a SR [28]. Few SRs investigated ICU and hospital LOS, preventing to deeply inspect the relationship between early discharges or avoided admissions and days of observation. Another difficulty in data collection regarded all patient-reported outcome measures (PROMs) such as disability and quality of life. However, it is known that PROMs collection can be particularly challenging in trauma care: low response of questionnaires administration, time-consuming and requirement of ad hoc training or specialist staff [49–51]. Despite barriers to PROMs collection in trauma registries, policymakers should address these challenges as PROMs have the potential to inform and guide patient-centred care and clinical decision-making in trauma care.

## Conclusion

The results of the present overview show that the application of a structured approach using a checklist including non-routine WBCT after initial assessment in selected patients during trauma resuscitation can offer positive patient- and process-related outcomes. Clinicians should consider these findings in the light of their clinical context, particularly in terms of balance between harm and benefits, volume of patients in their facilities, need for timely management, and the cost of WBCT.

## Supplementary Information

**Additional file 1**. Supplementary Information. **Appendix A**. Research question and search strategy;**Appendix B**. Modified Rand Delphi Process for prioritization of critical, important and unimportant outcomes;**Appendix C**. Primary studies and characteristics;**Appendix D**. Internal validity of systematic reviews;**Appendix E**. Certainty of Evidence.

## Data Availability

The data sets generated and/or analysed during the current study are available in the Open Science Framework repository at the following link: https://osf.io/2s68r/.

## Abbreviations

ABCDE: Airway, breathing, circulation, disability, exposure
AMSTAR 2: Assessing the Methodological Quality of Systematic Reviews
aOR: Adjusted odds ratio
ATCN: Advanced trauma course for nurses
ATLS: Advanced trauma life support
CCA: Corrected cover area
CT: Computed Tomography
CI: Confidence intervals
CoE: Certainty of evidence
eFAST: Extended focussed assessment sonography
ED: Emergency department
ER: Emergency room
ETC: European trauma course
GRADE: Grading of recommendations assessment, development and evaluation
ICU: Intensive care unit
IQR: Interquartile range
ISS: Injury severity score
LOS: Length of stay
MD: Mean difference
MOF: Multiple organ failure
MODS: Multiple organ dysfunction syndrome
OR: Odds ratio
PRISMA: Preferred reporting items for systematic reviews and meta-analyses
PICO: Patient, intervention, comparison and outcome
PTC: Prehospital trauma care
RCT: Randomized controlled trials
RoB: Cochrane risk of bias
RR: Relative risk
SMD: Standardized mean difference
SNLG: Sistema Nazionale Linee Guida
SR: Systematic review
WBCT: Whole-body computer tomography
WHO: World Health Organization
