# Supplementary material for: Structured approach with primary and secondary survey for major trauma care: an overview of reviews
Source: World J Emerg Surg. 2023 Jan 4;18:2. doi: 10.1186/s13017-022-00472-6 (PMC9814503; doi:10.1186/s13017-022-00472-6)
Supplement: Supplementary file 1 — Additional file 1. Supplementary Information. Appendix A. Research question and search strategy;Appendix B. Modified Rand Delphi Process for prioritization of critical, important and unimportant outcomes;Appendix C. Primary studies and characteristics;Appendix D. Internal validity of systematic reviews;Appendix E. Certainty of Evidence. [file 13017_2022_472_MOESM1_ESM.docx]

**Additional file 1**

[**Appendix A.** Research question and search strategy 1](#_Toc121865270)

[**Table S1. PICO Question** 2](#_Toc121865271)

[**Appendix B.** Modified Rand Delphi Process for prioritization of critical, important and unimportant outcomes 4](#_Toc121865272)

[**Appendix C.** Primary studies and characteristics 6](#_Toc121865273)

[**Comparison 1** 6](#_Toc121865274)

[**Table S1. Primary studies of SR across outcomes** 6](#_Toc121865275)

[**Comparison 2** 7](#_Toc121865276)

[**Table S2. Primary studies of SRs overall mortality** 7](#_Toc121865277)

[**Table S3. Primary studies of SRs mortality 24h** 8](#_Toc121865278)

[**Table S4. Primary studies of SRs ICU LOS** 9](#_Toc121865279)

[**Table S5. Primary studies of SRs complication** 10](#_Toc121865280)

[**Table S6. Primary studies of SRs hospital LOS** 11](#_Toc121865281)

[**Table S7. Primary studies of SRs time in ED/ER** 12](#_Toc121865282)

[**Appendix D.** Internal validity of systematic reviews 13](#_Toc121865283)

[**Table S1. AMSTAR 2** 13](#_Toc121865284)

[**Appendix E.** Certainty of Evidence 14](#_Toc121865285)

[**Table S1.** Certainty of Evidence of Structured approach versus clinical examination 14](#_Toc121865286)

[**Table S2.** Certainty of Evidence of WBCT verus no-WCBT 14](#_Toc121865287)

# **Appendix A.** Research question and search strategy

Review question: Is clinical and cost effective following a structured approach compared to only clinical examination to care in children, young people and adults who have suffered a suspected major chest and abdominal trauma?

| **Population** | Children, young people and adults who have experienced a suspected major trauma |
| --- | --- |
| **Intervention** | Structured/coordinated clinical approach as:  ATLS  ETC  ABCDE  Other clinical protocols or approach |
| **Comparison** | Not structured or coordinated approach as:  Only clinical examination |
| **Outcomes** | 1. 24 h Mortality 2. Overall Mortality (30 days) 3. ICU LOS (length of stay) 4. Complication 5. Adherence 6. Disability 7. Hospedalization LOS 8. Time spent 9. Radiation dose   Population size and directness:  No limitations on sample size  Studies with indirect populations will not be considered. |
| **Study design** | Systematic Reviews |
| **Exclusion** | People with a major trauma resulting from burns  Databases: Medline, Embase, the Cochrane Library  Date: All years  Study designs: Systematic Reviews |
| **Analysis** | Stratify by age: children (0-17 years), adults (18 and over)  Subgroups if between study heterogeneity:  Children by: infants (< 1 year), younger child (1-12 years) and older child / young person (12 years and older) |

## **Table S1. PICO Question**

**PUBMED**

POPULATION

| (trauma* or polytrauma*).ti,ab. |
| --- |
| ((serious* or severe* or major or life threaten*) adj3 (accident* or injur* or fall*)).ti,ab. |
| multiple trauma/ |
| wounds, gunshot/ or wounds, stab/ or accidents, traffic/ or accidental falls/ or blast injuries/ or accidents, aviation/ |
| ((motor* or motorbike* or vehicle* or road or traffic or car or cars or cycling or bicycle* or automobile* or bike* or head on or pile up) adj3 (accident* or crash* or collision* or smash*)).ti,ab. |
| (mvas or mva or rtas or rta).ti,ab. |
| (stabbed or stabbing or stab or gunshot* or gun or gunfire or firearm* or bullet* or knife* or knives or dagger).ti,ab. |
| or/1-7 |

INTERVENTION

((‘Primary Survey‘ [tiab] OR ‘Secondary Survey‘[tiab] OR ATLS[tiab] OR ‘Advanced trauma life support’[tiab] OR ETC[tiab] OR ‘European Trauma Course’[tiab] OR ‘Airway Breathing Circulation Disability Exposure’[tiab] OR ‘ABCDE’[tiab] OR 'ABCDE approach'[tiab]))

# **Appendix B.** Modified Rand Delphi Process for prioritization of critical, important and unimportant outcomes

ROUND I-II and final meeting

Prioritization outcomes

In the following questions concerning the question of interest we will ask you to evaluate the importance of the various outcomes for decision-making. Think about the impact that interventions in comparison could have on patients who have (or may have) Major Trauma.

***Tips & Reminders***

1. assess the importance of outcomes for decision-making in the context of the topic;

2. Assess the importance of outcomes regardless of your belief in the effects of interventions on

such outcomes;

3. Use your best knowledge of how important these results would be for people with

major trauma;

4. You can rate outcomes as equally important (no need to rank the order).

***Tasks***

Assess the relative importance of outcome for decision-making using a scale of 1 to 9. The meaning of the evaluations are:

1–3 are of little or no importance to decision-making

4–6 are important, but not critical to decision-making

7–9 are critical to decision-making.

NA not applicable (abstention vote)

Note: The same score can be attributed to more than one outcome of interest.


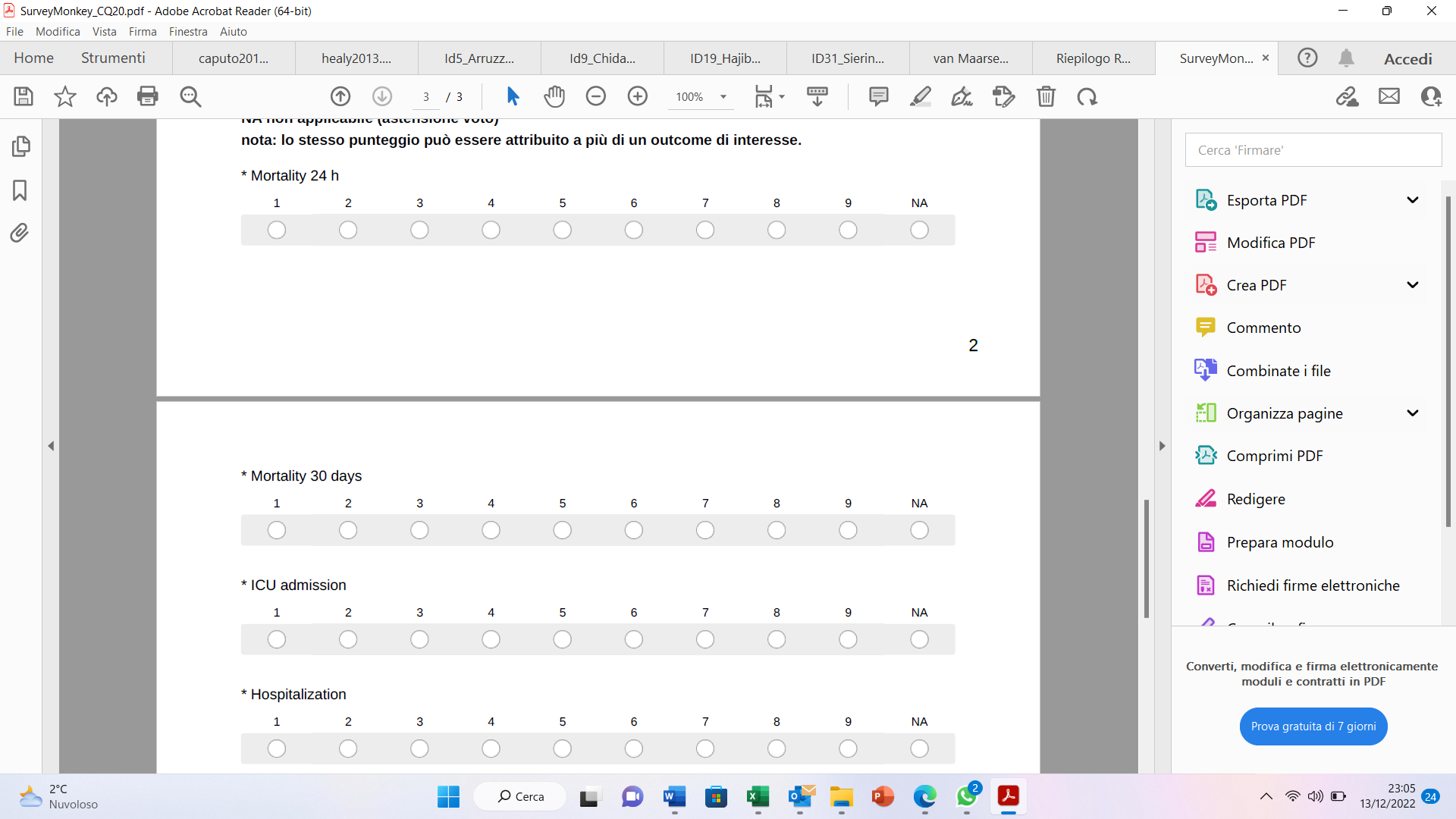


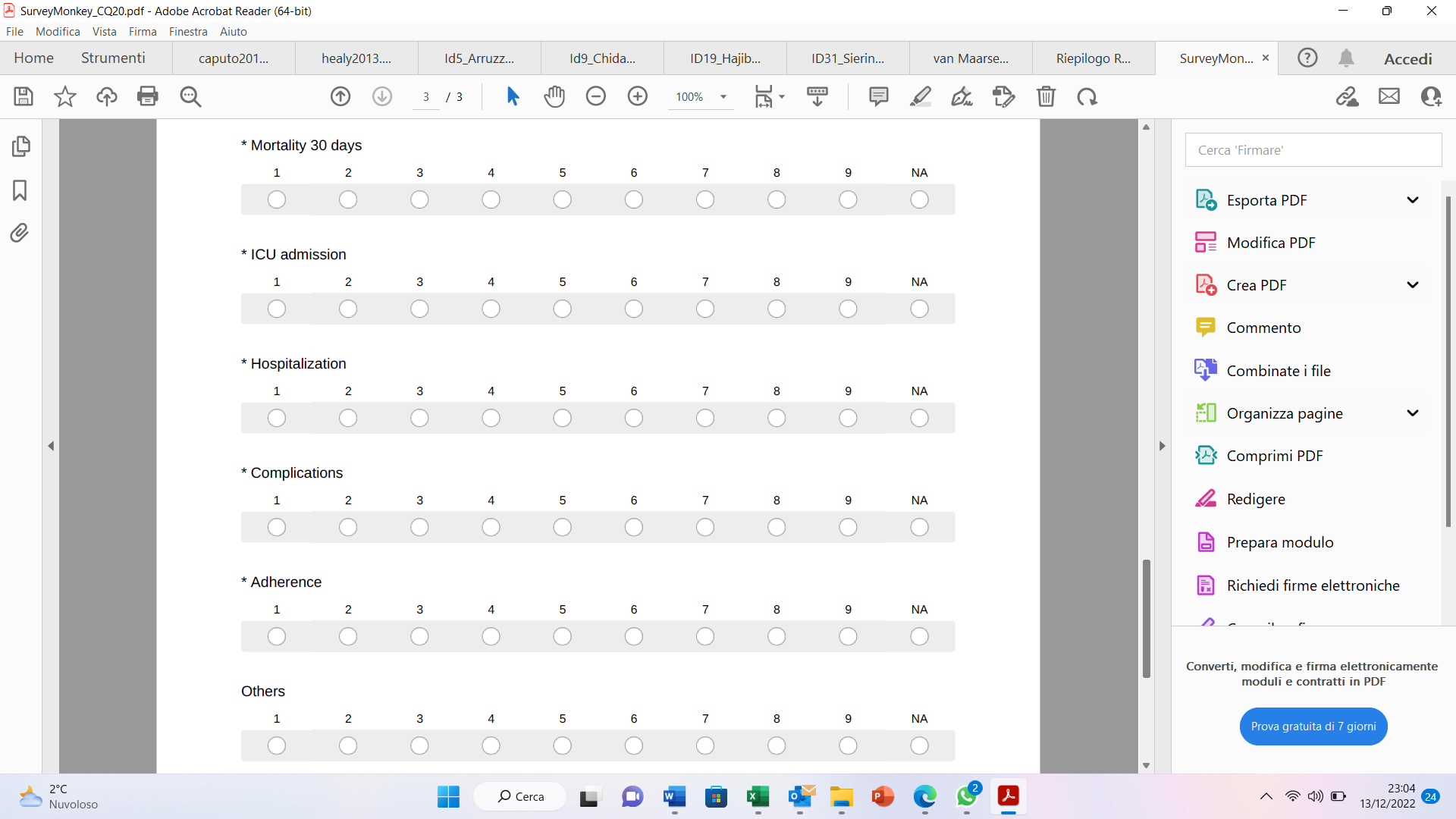


Finally, after two delphi rounds, the panel added few outcomes and assess the relative importance of outcome. The final list of critical and important outcomes voted were:

- **Critical**: overall mortality, mortality at 24 h, Intensive Care Unit (ICU) admission/ ICU length of stay (LOS) , complications (e.g., Multiple organ failure [MOF], multiple organ dysfunction syndrome [MODS], missed injuries), adherence, disability;
- **Important:** hospital admission/ hospital LOS, time in ED, time in ER, radiation dose.

# **Appendix C.** Primary studies and characteristics

## **Comparison 1**

## **Table S1. Primary studies of SR across outcomes**

| **SR** | **Primary studies** | **Overall mortality (30 days)** | **Mortality at 24 hours** | **ICU Admission/ ICU LOS** | **Complication/missed injuries** | **Adherence** | **Disability (ie. GOS)** | **Hospital admission/ Hospital LOS** | **Time in ED/ER** | **Radiation dose** |
| --- | --- | --- | --- | --- | --- | --- | --- | --- | --- | --- |
| **Van Maarseveen 2020** | Kelleher 2014a Kelleher 2014b |  |  |  |  | x |  |  |  |  |
|  | Lashoher 2016 | x |  |  | x | x |  |  |  |  |

GOS=Glasgow Outcome Scale GOS; LOS=Lenght of Stay

## **Comparison 2**

## **Table S2. Primary studies of SRs overall mortality**

*in light blue studies included in the meta-analysis, in white studies included in SRs.

## **Table S3. Primary studies of SRs mortality 24h**

## **Table S4. Primary studies of SRs ICU LOS**

## **Table S5. Primary studies of SRs complication**

## **Table S6. Primary studies of SRs hospital LOS**

## **Table S7. Primary studies of SRs time in ED/ER**

# **Appendix D.** Internal validity of systematic reviews

## **Table S1. AMSTAR 2**

|  | **Van Maarseveen 2018** | **Jiang 2013** | **Caputo 2013** | **Sierink 2012** | **Chidambaram 2017** | **Hajibandeh 2015** | **Van Vugt 2013** | **Healy 2013** | **Arruzza 2020** |
| --- | --- | --- | --- | --- | --- | --- | --- | --- | --- |
| **overall quality** | **low** | **Critically low** | **Critically low** | **Critically low** | **Critically low** | **Critically low** | **NA** | **low** | **Critically low** |
| 1-Question and inclusion | yes | yes | no | no | yes | no | yes | yes | yes |
| 2-Protocol | no | no | no | no | no | no | yes | no | no |
| 3-Study design | yes | no | no | no | no | no | no | no | no |
| 4-Comprehensive search | yes | yes | partial yes | yes | yes | no | yes | yes | yes |
| 5-Study selection | yes | yes | yes | yes | yes | yes | yes | no | yes |
| 6-Data extraction | yes | yes | yes | yes | yes | yes | yes | no | yes |
| 7-Excluded studied justification | no | no | no | no | no | no | yes | no | no |
| 8-Included studied details | yes | yes | no | yes | yes | yes | NA | yes | yes |
| 9-Risk of Bias | yes | no | no | yes | yes | yes | NA | yes | yes |
| 10-Source of funding of included studies | no | no | no | no | no | no | NA | no | no |
| 11-Appropriate statistical methods for analysis | yes | yes | yes | no | no | no | yes | yes | no |
| 12-Rob on meta-analyses | NA | no | no | no | no | no | NA | no | no |
| 13-Rob on individual studies | yes | no | no | yes | yes | no | NA | yes | no |
| 14-Explanation for heterogeneity | NA | no | yes | no | yes | no | NA | yes | yes |
| 15-Publication bias | NA | yes | yes | no | yes | no | NA | yes | yes |
| 16-Conflict of interest | yes | yes | yes | yes | yes | yes | yes | yes | yes |
|  |  |  |  |  |  |  |  |  |  |
| **total "no" critical (out of 7)** | **1** | 4 | 4 | 4 | **3** | 6 | NA | 1 | 4 |
| **total "no" NON-critical** | 1 | 4 | 5 | 5 | **3** | **5** | 1 | 5 | 3 |

# **Supplement E.** Certainty of Evidence

## **Table S1.** Certainty of Evidence of Structured approach versus clinical examination

| **COMPARISON**  structured approach vs  clinical examination | **Imprecision** | **Risk of bias * (study quality)** | **Inconsistency** | **Risk of bias**  **(review quality AMSTAR**)** | **LEVEL of EVIDENCE** |
| --- | --- | --- | --- | --- | --- |
| ***Overall mortality*** |  |  |  |  |  |
| **van Maarseveen 2020** | <200 | high RoB (-1) | >75%, SERIOUS (-1) | 3/4 YES, SERIOUS (-1) | LOW |
| ***Complication (MOF/MOD/missed injuries)*** |  |  |  |  |  |
| **van Maarseveen 2020** | <200 | high RoB (-1) | >75%, SERIOUS (-1) | 3/4 YES, SERIOUS (-1) | LOW |
| ***Adherence*** |  |  |  |  |  |
| **van Maarseveen 2020** | <200 | high RoB (-1) | >75%, SERIOUS (-1) | 3/4 YES, SERIOUS (-1) | LOW |

## **Table S2.** Certainty of Evidence of WBCT verus no-WCBT

| **COMPARISON**  WBCT vs non-WBCT | **Imprecision** | **Risk of bias * (study quality)** | **Inconsistency** | **Risk of bias**  **(review quality AMSTAR**)** | **LEVEL of EVIDENCE** |
| --- | --- | --- | --- | --- | --- |
| ***Overall mortality*** |  |  |  |  |  |
| Chidambaram 2017 | >200 | high RoB (-1) | >75%, SERIOUS (-1) | 3/4 YES, SERIOUS (-1) | LOW |
| Hajibandeh 2015 | >200 | high RoB (-1) | >75%, SERIOUS (-1) | 2/4 YES, SERIOUS (-2) | LOW |
| Arruzza 2020 | >200 | high RoB (-1) | >75%, SERIOUS (-1) | 3/4 YES, SERIOUS (-1) | LOW |
| Sierink 2012 | >200 | high RoB (-1) | <75%, NO SERIOUS | 3/4 YES, SERIOUS (-1) | MODERATE |
| Caputo 2013 | >200 | high RoB (-1) | >75%, SERIOUS (-1) | 2/4 YES, SERIOUS (-2) | LOW |
| Jiang 2013 | >200 | high RoB (-1) | >75%, SERIOUS (-1) | 3/4 YES, SERIOUS (-1) | LOW |
| Healy 2013 | >200 | high RoB (-1) | >75%, SERIOUS (-1) | 3/4 YES, SERIOUS (-1) | LOW |
| ***24-h mortality*** |  |  |  |  |  |
| Chidambaram 2017 | >200 | high RoB (-1) | >75%, SERIOUS (-1) | 3/4 YES, SERIOUS (-1) | LOW |
| Arruzza 2020 | >200 | high RoB (-1) | <75%, NO SERIOUS | 3/4 YES, SERIOUS (-1) | MODERATE |
| ***ICU LOS*** |  |  |  |  |  |
| Chidambaram 2017 | >200 | high RoB (-1) | >75%, SERIOUS (-1) | 3/4 YES, SERIOUS (-1) | LOW |
| Arruzza 2020 | >200 | high RoB (-1) | >75%, SERIOUS (-1) | 3/4 YES, SERIOUS (-1) | LOW |
| Jiang 2013 | >200 | high RoB (-1) | >75%, SERIOUS (-1) | 3/4 YES, SERIOUS (-1) | LOW |
| ***Complication (MODS/MOF)*** |  |  |  |  |  |
| Arruzza 2020 | >200 | high RoB (-1) | >75%, SERIOUS (-1) | 3/4 YES, SERIOUS (-1) | LOW |
| Jiang 2013 | >200 | high RoB (-1) | >75%, SERIOUS (-1) | 3/4 YES, SERIOUS (-1) | LOW |
| ***Radiation dose*** |  |  |  |  |  |
| Arruzza 2020 | >200 | high RoB (-1) | <75%, NO SERIOUS | 3/4 YES, SERIOUS (-1) | MODERATE |
| ***Hospedalization LOS*** |  |  |  |  |  |
| Chidambaram 2017 | >200 | high RoB (-1) | <75%, NO SERIOUS | 3/4 YES, SERIOUS (-1) | MODERATE |
| Sierink 2012 | >200 | high RoB (-1) | <75%, NO SERIOUS | 3/4 YES, SERIOUS (-1) | MODERATE |
| Arruzza 2020 | >200 | high RoB (-1) | >75%, SERIOUS (-1) | 3/4 YES, SERIOUS (-1) | LOW |
| Jiang 2013 | >200 | high RoB (-1) | <75%, NO SERIOUS | 3/4 YES, SERIOUS (-1) | MODERATE |
| ***Time spent in ER*** |  |  |  |  |  |
| Chidambaram 2017 | >200 | high RoB (-1) | >75%, SERIOUS (-1) | 3/4 YES, SERIOUS (-1) | LOW |
| Sierink 2012 | >200 | high RoB (-1) | NR | 3/4 YES, SERIOUS (-1) | NA |
|  |  |  |  |  |  |
| ***Time spent in ED*** |  |  |  |  |  |
| Arruzza 2020 | >200 | high RoB (-1) | >75%, SERIOUS (-1) | 3/4 YES, SERIOUS (-1) | LOW |
| Healy 2013 | >200 | high RoB (-1) | >75%, SERIOUS (-1) | 3/4 YES, SERIOUS (-1) | LOW |
| Jiang 2013 | >200 | high RoB (-1) | >75%, SERIOUS (-1) | 3/4 YES, SERIOUS (-1) | LOW |

* Proportion of participants included in the pooled analysis judged to have low ROB for randomisation and observer blinding (RCT). Non-RCT high ROB

** items considered in the AMSTAR 2: 1. covering a priori research design, 2. search characteristics, 3. independence of study selection and 4. data extraction (Pollock 2016).

RoB: Risk of Bias
